# Quantification of Influenza Virus RNA in Aerosols in Patient Rooms

**DOI:** 10.1371/journal.pone.0148669

**Published:** 2016-02-05

**Authors:** Nancy H. L. Leung, Jie Zhou, Daniel K. W. Chu, Han Yu, William G. Lindsley, Donald H. Beezhold, Hui-Ling Yen, Yuguo Li, Wing-Hong Seto, Joseph S. M. Peiris, Benjamin J. Cowling

**Affiliations:** 1 WHO Collaborating Centre for Infectious Disease Epidemiology and Control, School of Public Health, Li Ka Shing Faculty of Medicine, The University of Hong Kong, Pokfulam Road, Hong Kong SAR, China; 2 Centre of Influenza Research, Li Ka Shing Faculty of Medicine, The University of Hong Kong, Pokfulam Road, Hong Kong SAR, China; 3 Department of Mechanical Engineering, The University of Hong Kong, Pokfulam Road, Hong Kong SAR, China; 4 Allergy and Clinical Immunology Branch, Health Effects Laboratory Division, National Institute for Occupational Safety and Health, Centers for Disease Control and Prevention, Morgantown, West Virginia, United States of America; Johns Hopkins University - Bloomberg School of Public Health, UNITED STATES

## Abstract

**Background:**

The potential for human influenza viruses to spread through fine particle aerosols remains controversial. The objective of our study was to determine whether influenza viruses could be detected in fine particles in hospital rooms.

**Methods and Findings:**

We sampled the air in 2-bed patient isolation rooms for four hours, placing cyclone samplers at heights of 1.5m and 1.0m. We collected ten air samples each in the presence of at least one patient with confirmed influenza A virus infection, and tested the samples by reverse transcription polymerase chain reaction. We recovered influenza A virus RNA from 5/10 collections (50%); 4/5 were from particles>4 μm, 1/5 from 1–4 μm, and none in particles<1 μm.

**Conclusions:**

Detection of influenza virus RNA in aerosols at low concentrations in patient rooms suggests that healthcare workers and visitors might have frequent exposure to airborne influenza virus in proximity to infected patients. A limitation of our study was the small sample size. Further studies should be done to quantify the concentration of viable influenza virus in healthcare settings, and factors affecting the detection of influenza viruses in fine particles in the air.

## Introduction

Human influenza viruses spread with ease among susceptible humans in community settings such as households and school classrooms. Nosocomial transmission of influenza is a major concern because many hospitalized patients are more vulnerable to severe disease if infected with influenza. Influenza viruses can spread through respiratory droplets in the air, from a sneeze, cough or exhaled breath of an infected person, which then may be inhaled by another person [1]. The World Health Organization defines droplets as airborne particles with aerodynamic diameter >5μm [2], assuming droplets follow ballistic trajectories and generally do not travel further than 3 feet. Fine respiratory particles, or droplet nuclei generated from the rapid desiccation of the droplets upon expulsion into the environment, have diameter ≤5μm and can remain airborne for prolonged periods.

The role of fine particle aerosols in influenza transmission remains controversial, although viable influenza virus in aerosols has been detected in coughs and exhaled breath from infected persons [1, 3], can transit rooms [4], and can cause infections in humans [5]. Traditionally, large droplets have been thought to be the predominant mode of influenza transmission [6], and infection control guidelines recommend a spacing of ≥3 feet between beds [2]. In recent years, more evidence has arisen supporting an important role of aerosols in influenza transmission [7]. The objective of our study was to determine whether influenza viruses could be detected in fine particles in hospital rooms.

## Materials and Methods

### Study design

**Fig 1:**
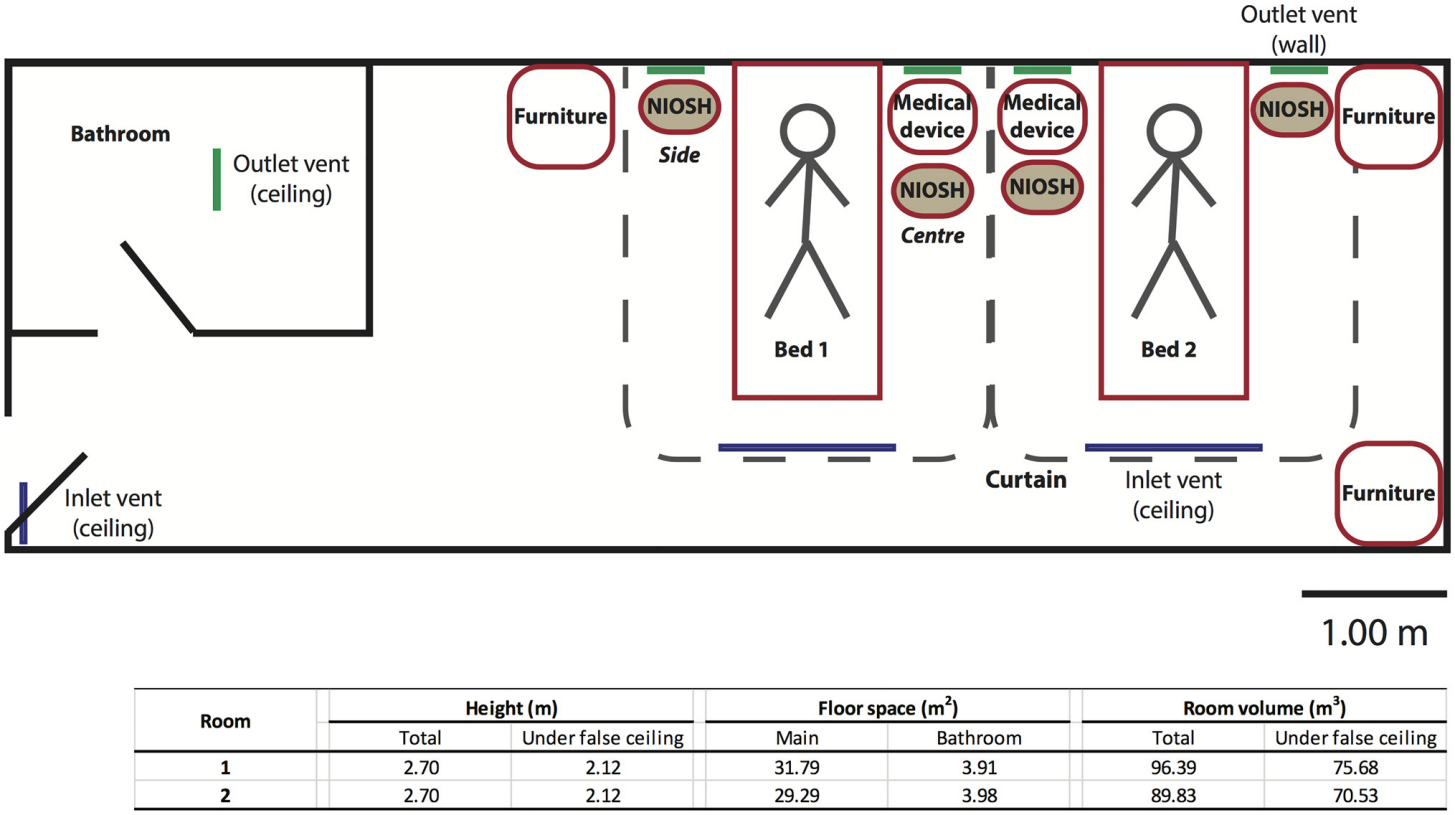
The layout of the patient rooms in which the air sampling for influenza A virus was conducted. The figure shows the usual positions for which the furniture, medical devices and the stationary air sampling device are located in one of the two patient rooms where the air sampling was conducted, although they are freely to be moved around (border in red). The other inpatient room is the mirror image of the first. For example if Bed 1 was the sampling bed, the air sampling device could be located at either ‘Centre’ or ‘Side’ as illustrated in the diagram. The dimensions of the two patient rooms where the air samplings were conducted were also provided. NIOSH: the air sampling device; Blue line: inlet vent; Green line: outlet vent.

We sampled air in two 2-bed isolation rooms on an adult infectious disease ward with patients generally admitted for acute respiratory illnesses, in a large private hospital in Hong Kong during the winter influenza season of 2014–15. The rooms had a design room ventilation rate of 12 air changes per hour (ACH). The layout of the rooms is shown in Fig 1.

On each sampling period, we positioned a stationary device in the patient room next to the bed of a patient with suspected or confirmed influenza virus infection (‘sampling bed’), and sampled the air for four hours continuously. One stationary air sampling device was used for each period of the air sampling. The device consisted of one or two two-stage cyclone air samplers (set at height 1.5m and 1.0m, which represent the mouth heights of a standing and sitting adult respectively) and a meter that recorded temperature and relative humidity (at height 1.3m) every four minutes. As shown in Fig 1, the NIOSH samplers were located either between the two beds but closer to the sampling bed (‘Centre’), on the side of the sampling bed (‘Side’), or were relocated during the period of the sampling as observed at the 0^th^, 2^nd^ and 4^th^ hour during the sampling period. The NIOSH samplers were placed in the range of 0.8m (when placed next to the sampling bed) to 3.2m (when placed on the further side of the other bed) from the patient’s head. A cloth curtain, hanging from a few inches from the ceiling to a few inches to the floor, was installed for each bed which may or may not enclose the air sampling device during the sampling, the information of which was provided in S1 Table (‘Curtain’). Air was collected at 3.5L/minute into three size fractions: >4μm (collected in a 15ml tube), 1–4μm (1.5ml tube) and <1μm (by a polytetrafluoroethylene (PTFE) membrane filter with 3.0μm pore size). The air samplers were developed by the US National Institute for Occupational Safety and Health (NIOSH) [8, 9]. For five sampling runs, an extra air sampler (set at height 0.8m) unconnected to a pump was added to the device as a negative control. After each collection, the 15ml and 1.5ml tubes were detached and 1 ml of viral transport medium (VTM) were added. The filter was removed and immersed in 1mL of VTM inside a 5ml tube. All the tubes were then transported to the laboratory at 4°C, vortexed, and the VTM was aliquoted and stored at -80°C for subsequent laboratory analysis. New sampling tubes and filters were used and the samplers and other equipment were disinfected between uses. The tripod, air tubing, sound-proof box and the meter were disinfected with Med-Clean (M&W International Ltd., Hong Kong), the NIOSH air samplers with 2% Citranox (Alconox Inc, NY, USA), and the filter cassettes were autoclaved. In addition, we collected basic information on the patients occupying the rooms, including laboratory confirmation of influenza A or B virus infections in these patients either by a rapid influenza chromatographic immunoassay or a multiplex PCR-based respiratory panel by the hospital laboratory. The position of the samplers and other relevant information was recorded at time 0, 2 and 4 hours during the sampling period.

### Ethical approval

The Institutional Review Board of The University of Hong Kong, and the Clinical and Research Ethics Committee of Hong Kong Baptist Hospital approved this study. All patients provided verbal informed consent; written consent was deemed unnecessary and waived by both Institutional Review Boards, given the environmental sampling approach and the limited information obtained from each patient retrospectively that did not include any personal information.

### Laboratory methods

For each air sample of 1ml collected, 300 μl were used for total RNA extraction and eluted to 25 μl, and 4 μl of the eluent was tested by quantitative RT-PCR for the presence of influenza viral particles using methods as previously described [10]. The limit of detection of the RT-PCR is 208 copies per 1ml original sample, and we considered samples with clear reaction signal growth curve with Ct values ≤40 to be positive for influenza.

### Statistical analysis

We described the concentration of influenza virus RNA detected in the air, patients’ demographics and diagnoses, and the summary measures of temperature and humidity by each period. The geometric means and SDs for the concentration of influenza virus in the air (copies/m^3^ air) were estimated by imputing the samples with undetectable level of virus as 1 copies/m^3^ air. We investigated the correlations by Spearman’s rank correlation coefficient (ρ) and the significance of independence by Mann-Whitney tests. All analyses were conducted with R version 3.2.0.

## Results

From 16 December 2014 through 9 February 2015, we performed air sampling on 16 periods, with 12 periods having two samplers and 4 periods just one sampler, for a total of 28 sets of air samples of three size fractions (S1 Table). Fig 1 shows the layout of the patient rooms including the locations of the inlet and outlet ventilation as well as where the samplers could be placed. No nebulizer therapy was performed on any of the patients during the 16 sampling periods.

On 12 periods at least one patient in the room had laboratory-confirmed influenza virus infection: 10 periods (17 sets of air samples) with laboratory-confirmed influenza A virus infection, and 3 periods (4 sets of air samples) with laboratory-confirmed influenza B virus infection (both a patient with laboratory-confirmed influenza A infection and another patient with influenza B infection were present in one period). Influenza B viruses were not recovered in any of the air samples from the 3 periods. We recovered influenza A virus from at least one of the size fractions in at least one air sampler in 5/10 (50%) periods (Table 1). We recovered mean 162 (SD 1.9) copies/m^3^ air of influenza A virus from 4/17 (24%) samples of the >4μm fraction, 144 copies/m^3^ air from 1/17 (6%) sample of the 1–4μm fraction, and no virus from the <1μm fraction of the air samples. 3/5 (60%) of the positive results were obtained from rooms in which the patient with confirmed influenza A virus infection was located in the bed that was farther from the sampler.

**Table 1:** Recovery of influenza A virus RNA in air in 2-bed inpatient rooms with at least one patient with laboratory-confirmed influenza A virus infection.

		Distance (m) of device from		Influenza A virus recovered in air (copies/m^3^ air)						Laboratory confirmation of influenza	
				Sampler at 1.5m			Sampler at 1.0m				
Identifier	Position of device	Sampling bed	Other bed	particles	particles	particles	particles	particles	particles	Sampling bed	Other bed
				>4 μm	1–4 μm	<1 μm	>4 μm	1–4 μm	<1 μm		
1	-	-	-	0	0	0	383	0	0	Neg	A(H3)
2	Centre	0.85	1.55	166	0	0	-	-	-	A(H3)	-
3	Side	0.90	3.20	94	0	0	-	-	-	-	A(H3)
4	Side	0.90	3.20	105	0	0	0	0	0	Neg	A(H3)
5	Side	1.00	3.20	0	0	0	0	144	0	A(H3)	-
6	Centre	0.80	1.60	0	0	0	0	0	0	A(H3)	A(H3)
7	Side	1.00	3.20	0	0	0	-	-	-	A(H3)	A(H3)
8	Changed	-	-	0	0	0	0	0	0	A(H3)	B
9	Centre	0.85	1.55	0	0	0	0	0	0	A(H3)	-
10	Centre	0.85	1.55	0	0	0	0	0	0	A(H3)	Neg

Footnotes: For ease of reference, the patients were ranked in the table as follows in decreasing priority: descending order of virus recovery or not in the air samples, descending order of the number of patients with laboratory-confirmed influenza infection in the patient room, and in chorological order of the air sampling. “Position of device”: the air sampling device was located either between the two beds but closer to the sampling bed (‘Centre’), on the side of the sampling bed (‘Side’), or was relocated during the period of the sampling (‘Changed’) as observed at the 0^th^, 2^nd^ and 4^th^ hour during the sampling period. Wherever possible, the distance of the air sampling device from the sampling bed and the other bed were also given. “Influenza A virus recovered in air”: Concentration of influenza A virus RNA recovered from samplers set at height 1.5m or 1.0m from the floor. Undetectable values were imputed as 0 copies/m^3^ air. “Laboratory confirmation of influenza”: laboratory confirmation of influenza A (‘A(H3)’) or B (‘B’) infection, or the absence of infection (‘Neg’), was done by PCR against influenza A or B viruses, or respiratory viral panel for a set of common respiratory viruses, on the patient’s nasopharyngeal swab.—: data not available.

In 4 sampling periods where none of the patients in the room had laboratory-confirmed influenza, we did not detect any influenza virus RNA in the air. The details of the patients and the rooms of all 16 periods are set out in S1 Table.

**Fig 2:**
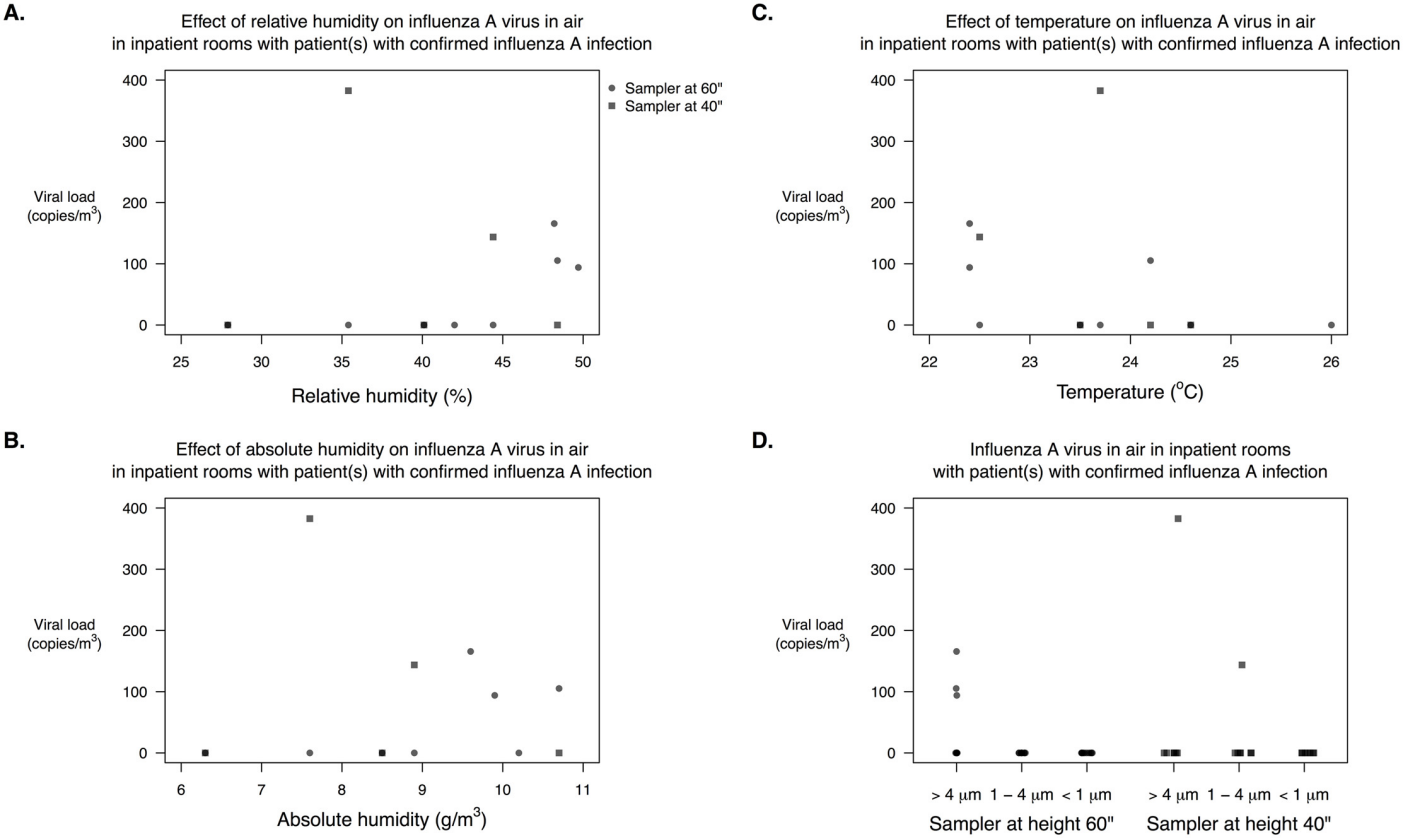
Scatter plots of the concentration of influenza virus RNA detected in the air (influenza virus RNA particles/m^3^ of air) versus relative humidity (A), absolute humidity (B), temperature (C) and the height of the sampler (D). Dot indicates the concentration of RNA copies of influenza virus recovered from a sampler set at 1.5 m from the floor, and square indicates that from a sampler set at 1.0 m from the floor.

We monitored temperature and relative humidity continuously in 8/10 (80%) sampling periods conducted in the presence of at least one patient with laboratory-confirmed influenza A virus infection. The range of mean temperature, relative humidity and absolute humidity for the 8 periods was 22.4–26.0°C, 27.9–49.7% and 6.3–10.7g/m^3^, with maximum standard deviation for individual periods as 1.1°C, 2.3% and 0.4g/m^3^ respectively. We investigated the correlations between the total concentration of influenza virus RNA in the air against the temperature, relative humidity and absolute humidity for each air sampler (Fig 2). None of the correlations were significant in the complete data set (temperature: ρ = -0.45, p-value = 0.13; relative humidity: ρ = 0.34, p-value = 0.25; absolute humidity: ρ = 0.20, p-value = 0.51), but the correlations against the temperature and relative humidity become significant when the outlier of the air sample with highest influenza virus RNA recovered was removed (temperature: ρ = -0.58, p-value = 0.05; relative humidity: ρ = 0.60, p-value = 0.04; absolute humidity: ρ = 0.42, p-value = 0.18). There was no significant difference between the total concentration of influenza virus RNA in the air and the height of the air sampler (complete data set: p-value = 0.90; outlier removed: p-value = 0.67).

**Fig 3:**
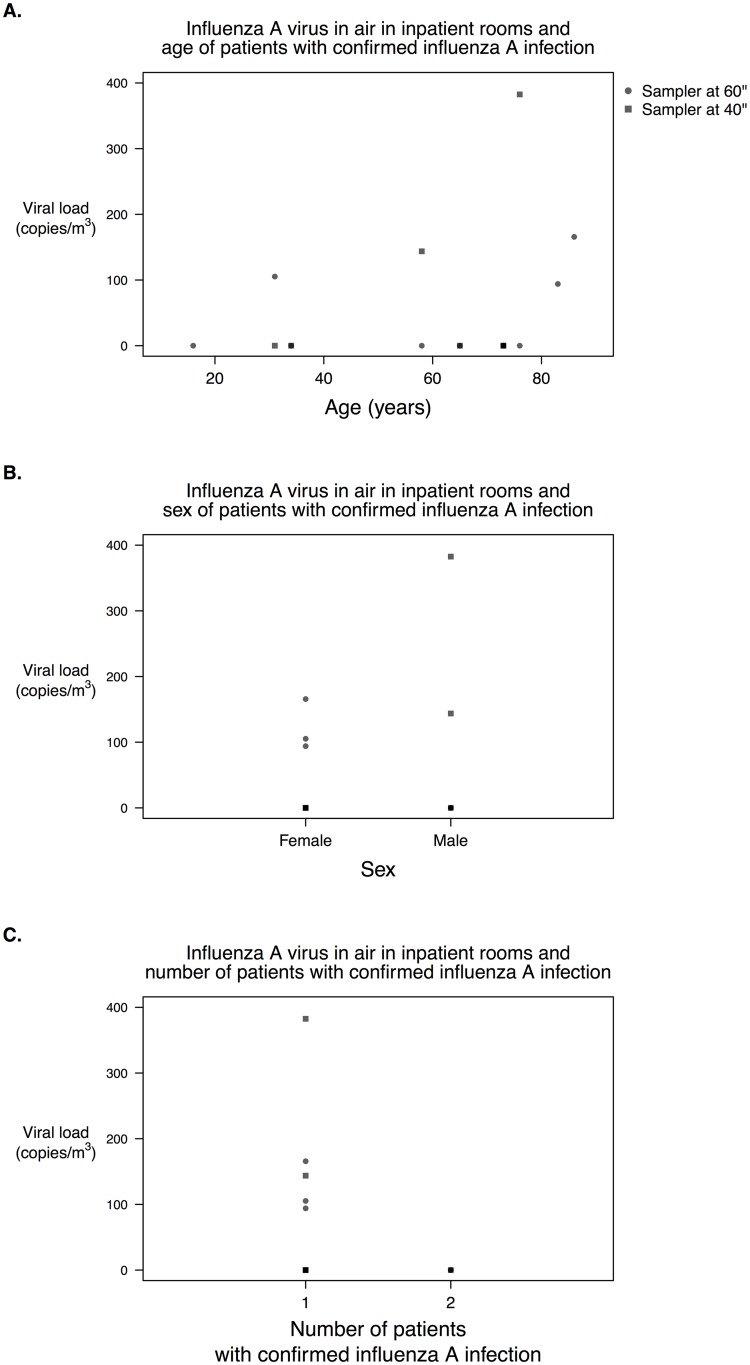
Scatter plots of the concentration of influenza virus RNA detected in the air versus (A) age and (B) sex of the patient with laboratory-confirmed influenza A infection, and (C) number of patients with laboratory-confirmed influenza A infection in the room. If there were more than one patient with laboratory-confirmed influenza A infection in the room, the data of the patient on the sampling bed was used. Dot indicates the concentration of RNA copies of influenza virus recovered from a sampler set at 1.5 m from the floor, and square indicates that from a sampler set at 1.0 m from the floor.

We investigated the correlations between the total concentration of influenza virus RNA in the air against the age and the number of patients with confirmed influenza A virus infection (Fig 3). On two periods when there were more than one infected patient in the room, the value of the patient nearer to the air sampler was used. The correlations were not significant (age: ρ = 0.34, p-value = 0.19; number of infected patients: ρ = -0.29, p-value = 0.25).

## Discussion

In patient rooms with patients with confirmed influenza virus infection, we detected influenza virus RNA in aerosols at low concentrations half of the times. This is likely to be the upper bound of the frequency of exposure since we measured only the concentration of total virus but not the infectious virus. In 80% of the positive sampling events (4/5), influenza viral RNA was detected in particles>4μm. The use of surgical face masks is recommended for both healthcare workers and visitors to hospitals in Hong Kong, and might reduce the risk of droplet transmission, although surgical masks are not adequate to protect against exposures to fine particle aerosols [1].

In this study we did not quantify the absolute risk of exposure to an infectious dose of influenza virus for a given exposure period. Further studies of this type with recovery of infectious virus would help to characterize the risk of exposure to influenza virus in aerosols in the respirable fraction that may induce more severe disease by initiating an infection in the lower respiratory tract [7], and might be used to estimate the risk of infection to infectious doses of influenza virus in air in healthcare settings during the influenza season.

Environmental factors such as temperature and humidity may affect influenza transmission and contribute to the seasonality of influenza epidemics, with efficient transmission demonstrated under dry conditions. Shaman *et al*. showed absolute humidity, rather than relative humidity, explained most of the variability in influenza virus survival in controlled conditions with temperature and humidity as the only varying factors [11]. Here we showed that influenza virus RNA recovery in the air was significantly associated with decreasing temperature and increasing relative humidity after one outlier was removed (Fig 2). There was an apparent positive association between virus recovery and humidity in the range of absolute humidity which is shown to affect the efficiency of transmission in guinea pigs (6–11 g/m^3^) [12], but with a stronger association with relative humidity than absolute humidity (Fig 2). A study with larger sample size would be needed to confirm this potential association. Humidity might affect virus recovery either by affecting the production of virus-laden fine particles via affecting the rate of evaporation and the final size of the particles [13], or the viability of the virions in the particles [11], but we did not find an association between increasing humidity and increasing virus recovery in larger particles among previous studies [4, 8, 14, 15].

Detection of influenza A virus RNA in 5/10 (50%) rooms was consistent with the presence of patients with influenza in these rooms, and it is highly likely that these patients were the source. Bischoff *et al*. showed that some patients emitted virus up to 32 times more than others [15], demonstrating the importance of identifying patient characteristics which may help to identify more infectious individuals. We were underpowered to identify a significant association between patients’ characteristics and virus recovered in air. Further studies with larger sample size and that include other possible factors, e.g. the time since illness onset, could be useful in identifying emitters.

Influenza virus RNA has been detected in air in areas with suspected or known sources of influenza virus in healthcare settings previously [8, 14, 15]. Lindsley *et al*. demonstrated 53% of the total influenza virus RNA detected in the air in a hospital emergency department [14], and 40–50% of the influenza A virus RNA detected in stationary samplers or personal samplers in an urgent care clinic was in particles ≤4 μm [8]. In a similar study to our own, Bischoff *et al*. sampled aerosolized influenza virus in patient rooms with influenza-infected patients for 20 minutes with three 6-stage Andersen samplers placing at 1, 3 and 6 feet from the patient’s head and also collected nasopharyngeal swabs from these patients [15]. They found aerosolized influenza virus in 43% of patients with confirmed influenza virus infection, and up to 89% of influenza-laden particles were fine particles <4.7 μm. They recovered influenza virus as far as 6 feet (1.8m) away from the patients with almost all recovered in the small particles. This is consistent with those findings, because we also detected influenza virus RNA in aerosols at low concentrations half of the times in patient rooms with patients with confirmed influenza virus infection although we could not confirm whether the virus was infectious using viral culture. Furthermore, we recovered influenza A virus RNA as far as 3m away from the influenza-infected patients in patient rooms with higher ventilation rate (12 ACH versus 6 ACH in Bischoff *et al*.). This suggests other patients sharing the same patient room, healthcare workers and visitors possibly have frequent exposure to airborne influenza virus in proximity to influenza infected patients, even in patient rooms which are designed to have high ventilation rates. In addition, we measured temperature and humidity during each air sampling period, and showed that influenza virus RNA recovery in the air was significantly correlated with temperature and relative humidity. On the other hand, we recovered 93% of the total virus in particles >4 μm and 7% in particles 1–4 μm. There are many factors that could explain this discrepancy in observing the majority of influenza virus RNA in air in larger rather than smaller particles, for example the different samplers used, the difference in environmental factors (temperature, humidity and ventilation rate) and the size of the patient rooms where the air sampling was conducted. Further studies would be needed to confirm and assess the extent of these factors in affecting the virus distribution over different size particles in the air.

There are a few limitations of our study. First, we did not attempt to culture virus since we expected that at least 4 log_10_ RNA copies per sample would be needed for successful isolation [1]. Other work has demonstrated that a small fraction of viral RNA in aerosols is infectious virus [1, 3]. Second, we did not collect detailed information from patients, such as the time since illness onset and the use of oseltamivir treatment. We plan to collect more detailed information from patients in a subsequent study. Third, we only sampled in 1–2 locations in each sampling period. In future studies it would be valuable to sample in a greater number of locations, and also measure airflow patterns and ventilation rates, to allow a more comprehensive risk assessment. Finally, we were underpowered to detect significant correlations between the total concentration of influenza A virus in the air and the temperature and humidity in the complete data set but were able to detect when the outlier was removed. The relatively stable temperature and humidity during the day in the patient rooms studied compared to that in other settings (e.g. households) would allow the assessment of the effect of the temperature and humidity on virus survival and on aerosol size distribution in the air.

In conclusion, we found influenza virus RNA in patient rooms, demonstrating the feasibility of this study design to contribute to risk assessment of nosocomial transmission of influenza. Further larger studies would be worthwhile, including the use of more samplers, collection of detailed data from individual patients, and perhaps detailed measurement of other environmental parameters to permit airflow modeling. It would also be valuable to conduct this type of study in rooms during the use of aerosol generating procedures, to quantify the additional risk posed to health care personnel by particular procedures.

## Supporting Information

S1 Table Details of the patients and the 2-bed patient rooms where the air samplings were conducted for all 16 periods. “Position of device”: the air sampling device was located either between the two beds but closer to the sampling bed (‘Centre’), on the side of the sampling bed (‘Side’), or was relocated during the period of the sampling (‘Changed’) as observed at the 0^th^, 2^nd^ and 4^th^ hour during the sampling period. Wherever possible, the distance of the air sampling device from the sampling bed and the other bed were also given. “Curtain”: the curtain around the sampling bed might be drawn to enclose the NIOSH sampler (‘Enclosed’), not drawn (‘Not enclosed’) or has changed from drawn to not drawn (or vice versa) (‘Changed’) during the sampling period. “Influenza A virus recovered in air”: Concentration of influenza A virus RNA recovered from samplers set at height 1.5m or 1.0m from the floor, or from negative control of which the air samplers (set at height 0.8m) were not connected to the air pumps. Undetectable values were imputed as 0 copies/m^3^ air, while the Ct values were shown in bracket for air samples which we considered positive. “Diagnosis”: the diagnosis of the patient at discharge (‘URTI’: upper respiratory tract infection). “Laboratory confirmation of influenza”: laboratory confirmation of influenza A (‘A(H3)’) or B (‘B’) infection, or the absence of infection (‘Neg’), was done by PCR against influenza A or B viruses, or respiratory viral panel for a set of common respiratory viruses, on the patient’s nasopharyngeal swab. “Environmental factors”: ‘Temperature’ (°C) and ‘relative humidity’ (%) were measured every four minutes during the sampling period by a meter set at height 1.3m. Absolute humidity, expressed as the mass of water vapor per cubic meter of air (g/m^3^), was calculated from the temperature and relative humidity measured. The mean and the standard deviation (‘SD’) of the three measures were presented for each collection. ‘Volume of the room (m^3^)’ under false ceiling was also presented for each collection.—: data not available.
(PDF)
