# Supplementary material for: Quantification of Influenza Virus RNA in Aerosols in Patient Rooms
Source: PLoS One. 2016 Feb 5;11(2):e0148669. doi: 10.1371/journal.pone.0148669 (PMC4743992; doi:10.1371/journal.pone.0148669)
Supplement: S1 Table — “Position of device”: the air sampling device was located either between the two beds but closer to the sampling bed (‘Centre’), on the side of the sampling bed (‘Side’), or was relocated during the period of the sampling (‘Changed’) as observed at the 0th, 2nd and 4th hour during the sampling period. Wherever possible, the distance of the air sampling device from the sampling bed and the other bed were also given. “Curtain”: the curtain around the sampling bed might be drawn to enclose the NIOSH sampler (‘Enclosed’), not drawn (‘Not enclosed’) or has changed from drawn to not drawn (or vice versa) (‘Changed’) during the sampling period. “Influenza A virus recovered in air”: Concentration of influenza A virus RNA recovered from samplers set at height 1.5m or 1.0m from the floor, or from negative control of which the air samplers (set at height 0.8m) were not connected to the air pumps. Undetectable values were imputed as 0 copies/m3 air, while the Ct values were shown in bracket for air samples which we considered positive. “Diagnosis”: the diagnosis of the patient at discharge (‘URTI’: upper respiratory tract infection). “Laboratory confirmation of influenza”: laboratory confirmation of influenza A (‘A(H3)’) or B (‘B’) infection, or the absence of infection (‘Neg’), was done by PCR against influenza A or B viruses, or respiratory viral panel for a set of common respiratory viruses, on the patient’s nasopharyngeal swab. “Environmental factors”: ‘Temperature’ (°C) and ‘relative humidity’ (%) were measured every four minutes during the sampling period by a meter set at height 1.3m. Absolute humidity, expressed as the mass of water vapor per cubic meter of air (g/m3), was calculated from the temperature and relative humidity measured. The mean and the standard deviation (‘SD’) of the three measures were presented for each collection. ‘Volume of the room (m3)’ under false ceiling was also presented for each collection.—: data not available. (PDF) [file pone.0148669.s001.pdf]

		Distance (m) of device from			Influenza A virus recovered in air (copies/m^3 air)								
					Sampler at 60"			Sampler at 40"			Negative control		
Identifier	Position of device	Sampling bed	Other bed	Curtain	particles >4 µm	particles 1-4 µm	particles <1 µm	particles >4 µm	particles 1-4 µm	particles <1 µm	particles >4 µm	particles 1-4 µm	particles <1 µm
1	-	-	-	Changed	0	0	0	383 (36.7)	0	0	-	-	-
2	Centre	0.85	1.55	Enclosed	166 (38.0)	0	0	-	-	-	-	-	-
3	Side	0.90	3.20	Enclosed	94 (38.9)	0	0	-	-	-	-	-	-
4	Side	0.90	3.20	Enclosed	105 (38.7)	0	0	0	0	0	-	-	-
5	Side	1.00	3.20	Changed	0	0	0	0	144 (38.2)	0	-	-	-
6	Centre	0.80	1.60	Enclosed	0	0	0	0	0	0	-	-	-
7	Side	1.00	3.20	Enclosed	0	0	0	-	-	-	-	-	-
8	Changed	-	-	Enclosed	0	0	0	0	0	0	0	0	0
9	Centre	0.85	1.55	Not enclosed	0	0	0	0	0	0	0	0	0
10	Centre	0.85	1.55	Not enclosed	0	0	0	0	0	0	-	-	-
11	-	-	-	Changed	0	0	0	0	0	0	0	0	0
12	-	-	-	Changed	0	0	0	0	0	0	0	0	0
13	-	-	-	Changed	0	0	0	0	0	0	0	0	0
14	Side	1.00	3.20	Enclosed	0	0	0	0	0	0	-	-	-
15	Side	1.00	3.20	Enclosed	0	0	0	-	-	-	-	-	-
16	Side	1.00	3.20	Enclosed	0	0	0	0	0	0	-	-	-

Patient's information							
At the sampling bed				At the other bed			
Age	Sex	Diagnosis	Laboratory confirmation of influenza	Age	Sex	Diagnosis	Laboratory confirmation of influenza
45	M	URTI	Neg	76	M	Influenza A	A(H3)
86	F	Influenza A	A(H3)	33	F	Bronchitis	-
28	F	Tonsilitis/ Pharyngitis	-	83	F	Influenza A	A(H3)
68	F	Pneumonia	Neg	31	F	Influenza A	A(H3)
58	M	Influenza A	A(H3)			No patient admitted	
34	M	Influenza A	A(H3)	12	M	Influenza A	A(H3)
16	F	Influenza A	A(H3)	72	F	Influenza A, Pneumonitis	A(H3)
73	F	Influenza A	A(H3)	11	F	Influenza B, Pneumonia	B
73	F	Influenza A	A(H3)			No patient admitted	
65	F	Influenza A	A(H3)	68	F	Pneumonia	Neg
-	-	Bronchitis, URTI	-	39	M	Pharyngitis, URTI	Neg
14	M	Syncope, URTI	Neg	93	M	Pneumonia	Neg
63	M	Pneumonia	Neg			No patient admitted	
53	M	Influenza B	B	56	M	Pneumonia	-
73	M	Pneumonia	Neg	-	-	-	-
79	M	Myositis, Influenza B	B	21	M	Bronchitis, Syncope	Neg

Volume of the room (m^3)	Environmental factors					
	Temperature (°C)		Relative humidity (%)		Absolute humidity (g/m^3 air)	
	Mean	SD	Mean	SD	Mean	SD
70.53	23.7	0.3	35.4	1.8	7.6	0.3
75.68	22.4	0.3	48.2	0.6	9.6	0.2
75.68	22.4	0.1	49.7	0.8	9.9	0.1
75.68	24.2	1.1	48.4	2.3	10.7	0.4
70.53	22.5	0.5	44.4	0.9	8.9	0.2
70.53	24.6	0.7	27.9	1.4	6.3	0.2
70.53	26	0.6	42	1.1	10.2	0.2
75.68	-	-	-	-	-	-
75.68	-	-	-	-	-	-
75.68	23.5	0.3	40.1	0.9	8.5	0.1
70.53	22.1	0.2	37.4	1.1	7.3	0.2
75.68	20.9	0.1	25.4	0.8	4.7	0.2
70.53	22.6	0.5	44	1.9	8.8	0.6
70.53	25.5	0.5	36.9	1.5	8.8	0.2
70.53	22.9	0.2	54.9	0.7	11.2	0.2
70.53	23.7	0.4	52.3	1.9	11.2	0.3
